# Ethical Issues in Breastfeeding and Lactation Interventions: A Scoping Review

**DOI:** 10.1177/08903344231215073

**Published:** 2023-12-12

**Authors:** Supriya Subramani, Rasita Vinay, Julian W. März, Michaela Hefti, Nikola Biller-Andorno

**Affiliations:** 1 Sydney Health Ethics, School of Public Health, Faculty of Medicine and Health, University of Medicine, Camperdown, NSW, Australia; 2 Institute of Biomedical Ethics and History of Medicine, University of Zurich, Zurich, Switzerland; 3 Family Larsson-Rosenquist Foundation, Frauenfeld, Switzerland

**Keywords:** breastfeeding, breastfeeding interventions, ethics, lactation, public health, scoping review

## Abstract

**Background:**

Infant feeding interventions that promote and support breastfeeding are considered important contributions to global public health. As these interventions often target private settings (e.g., individuals’ homes) and involve vulnerable populations (e.g., pregnant women, infants, and underprivileged families), a keen awareness of ethical issues is crucial.

**Research Aim:**

The purpose of this scoping review was to capture the key elements of the current ethical discourse regarding breastfeeding and lactation interventions.

**Method:**

A scoping review was conducted using Arksey and O’Malley’s (2005) methodology to identify the ethical issues of breastfeeding and lactation interventions as they are reflected in the scholarly literature published between January 1990 and October 2022. Abstracts (*N* = 3715) from PubMed, ScienceDirect, JSTOR and the Cochrane Database of Systematic Reviews were screened. The final sample consisted of 26 publications.

**Results:**

The recurring ethical issues identified in these studies were: the normative assumptions of motherhood; maternal autonomy and informed choice; information disclosure, balancing risks and benefits, and counseling practices; stigma and social context; ethics of health communication in breastfeeding campaigns; and the ethical acceptability of financial incentives in breastfeeding interventions.

**Conclusion:**

This review illustrated that, while a wide range of ethical arguments were examined, the emphasis has been primarily on accounting for mothers’ experiences and lactating persons’ choices, as well as achieving public health objectives relating to infant nutrition in breastfeeding interventions. To effectively and ethically implement breastfeeding and lactation interventions, we must consider the social, economic, and cultural contexts in which they occur. One key learning identified was that women’s experiences were missing in these interventions and, in response, we suggest moving beyond the dichotomous approach of individual health versus population health.

**Key Messages**

- An awareness of ethical issues in breastfeeding and lactation interventions is crucial given that they often target private settings and involve vulnerable populations. There is little current knowledge of the ethical issues associated with breastfeeding and lactation interventions.
- We identified two dominant sets of ethical issues, one centered around mothers’ experiences and one around children’s access to breastmilk from a public health perspective.
- The key learning is that women’s experiences are often not accounted for when designing and implementing the interventions, and we suggest moving beyond the dichotomous approach of individual autonomy versus population health in breastfeeding interventions.
- This review suggests that to implement breastfeeding and lactation interventions effectively and ethically, we must consider lactating persons’ experiences from a broad range of social, economic, and cultural contexts.

## Background

Public health interventions have raised ethical concerns that differ from clinical and biomedical research interventions. They have often focused on prevention aimed at individuals rather than the environment to protect, promote, and support health. Several frameworks and guidelines have been developed to help stakeholders involved in public health programs or interventions to reflect on ethical issues that might arise during the implementation, or any other stage of these interventions. The key ethical issues that have been given importance in public health interventions are the protection of human rights and health benefits, the potential for harm, the need for justice, social solidarity, and the prevention of stigmatization and marginalization to avoid threats to autonomy (Buchanan, 2019; Gopichandran et al., 2016; Have et al., 2010, 2013; Kass, 2001; Marckmann et al., 2015). Many scoping reviews that have attempted to map the ethical issues of breastfeeding interventions have so far been limited to specific interventions—for example, financial incentives (Hoskins et al., 2019; South et al., 2014)—or within the broader scope of nutrition related health interventions (Hurlimann et al., 2017). While the existing frameworks have addressed public health interventions more generally or ethical issues relating to other areas, there has been a lack of specific guidance for breastfeeding and lactation interventions.

Increased breastfeeding rates are an important public health objective, and early initiation and exclusive breastfeeding are the most effective interventions to reduce morbidity and mortality in infants and children (Victora et al., 2016). In 1990, by acknowledging the Innocenti Declaration, the World Health Organization (WHO) and United Nations Children’s Fund (UNICEF) declared breastfeeding—where the child receives milk directly from the breast or expressed humanmilk—a global health priority. They stated that all women should be enabled to practice exclusive breastfeeding and all infants should be fed exclusively on breastmilk from birth to 4–6 months of age (Labbok & Starling, 2012; WHO 2008). Since then, increasing breastfeeding rates have become a primary goal for local and global health organizations, with most breastfeeding and lactation interventions aimed at ensuring children’s right to adequate nutrition. Since the impetus of improving child and maternal health has been morally uncontroversial, ethical challenges that have emerged during the development and implementation of corresponding public health interventions have been easily overlooked.

We conducted a scoping review as part of a larger effort to develop an ethical framework for breastfeeding and lactation interventions. The purpose of this scoping review was to capture the key elements of the current ethical discourse regarding breastfeeding and lactation interventions.

## Method

### Design

This study was a scoping review. The Arksey and O’Malley (2005) review methodology provided a transparent way to explore and synthesize literature to identify and map the range of ethical issues in breastfeeding and lactation interventions as they were reflected in the scholarly literature to date. The findings have been presented on a conceptual map to display the most prominent themes, allowing us to identify the current gaps that require further research.

### Sample: Defining the Articles Reviewed

The Innocenti Declaration, which was endorsed by the WHO and UNICEF in 1990, emphasized exclusive breastfeeding as a global health priority. This focus led many local and global organizations to include breastfeeding as part of their public health policy and programs. We reflected on ethical issues in breastfeeding and lactation interventions since the beginning of these efforts by searching literature published between January 1990 and October 2022.

#### Abstract Review

**Figure 1.**
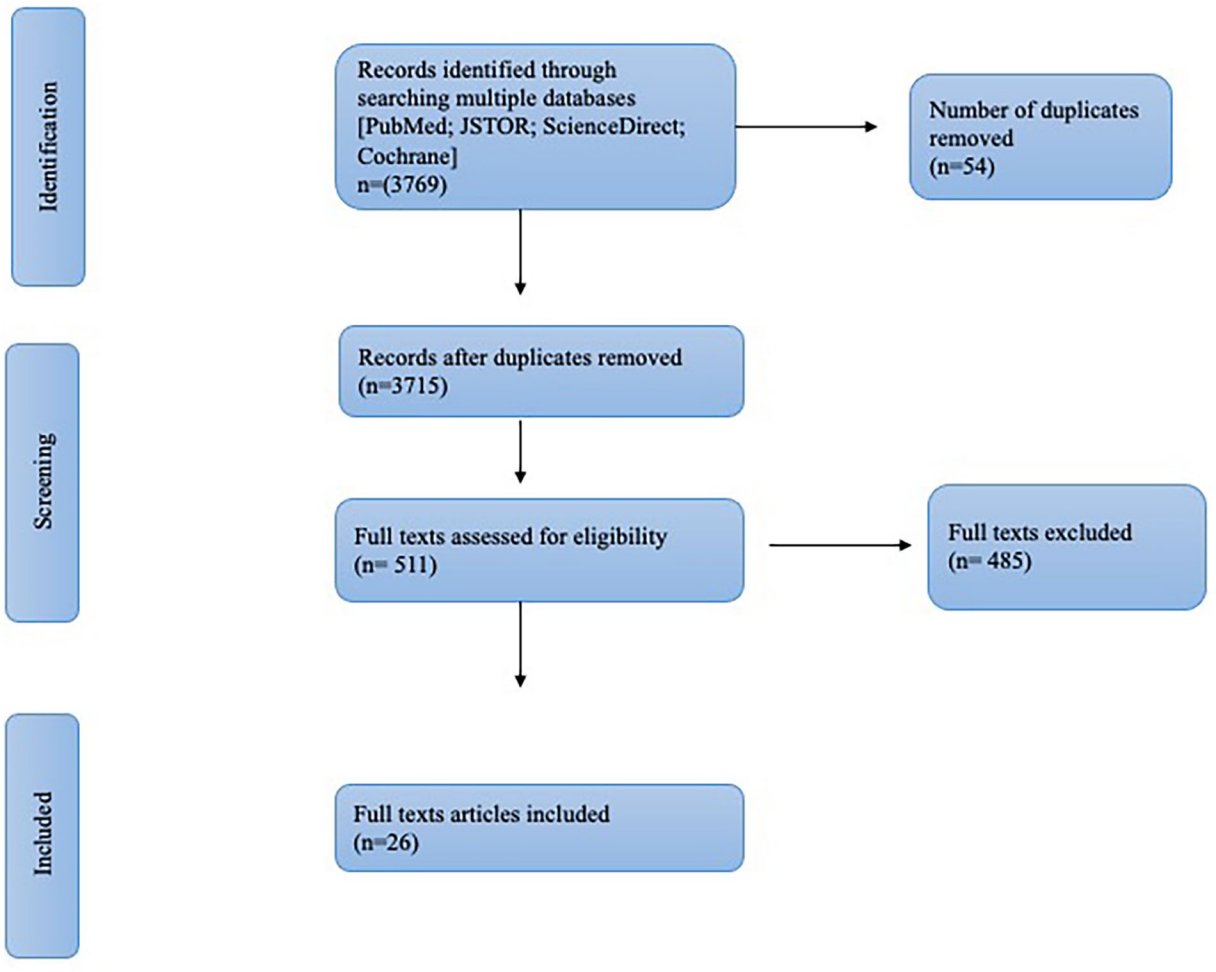
PRISMA Flow Chart.

Articles (*N* = 3769) were reviewed based on title and abstract. After removing duplicates and other miscellaneous articles (conference reports, front and back matter, and bibliography notes), 3715 articles were retained. The inclusion criteria for article eligibility were as follows: Intervention ethics studies that considered breastfeeding/infant feeding practices or articles explicitly describing ethical issues in breastfeeding, lactation, and infant feeding practices (even without using the words ethics or ethical in the title). Articles were excluded if they were from non-human studies, did not address ethical issues, focused only on breastfeeding and infant feeding interventions, included only research ethics, were on education in the study of ethics, and also editorials, reports, and short news interviews and commentaries and articles not in English. The PRISMA flow chart for the search strategy and results is presented in Figure 1 and Supplemental Table 1.

Three reviewers (SS, JM and RV) independently screened records. If the relevance of a study was unclear from the abstract, it was included for a full-text review. For the full-text review, 511 studies were included.

#### Full-text review

Articles (*n* = 511) were reviewed independently by the researchers (SS and RV). The first author (SS) further analyzed the full texts, and disagreements were resolved by SS, who had developed the inclusion criteria along with the PI (NBA) and other reviewers (RV and JM). As a result, 485 articles were excluded for the following reasons: (a) they only mentioned ethics in the context of ethics approval (or exemption of ethics approval) from a research ethics board; (b) they mentioned ethics only as part of the affiliation of the author (an institute/department of ethics) in the body of the text; and (c) breastfeeding was mentioned but the content did not engage with breastfeeding interventions and associated ethical issues. In the current scoping review, 26 studies were included as a result of this exercise and were critically analyzed by the first author (SS). A detailed statement of reflexivity is provided (see the online Supplemental Material).

### Data Collection: The Search Strategy and Process

The scoping review process was conducted between May 2021 and October 2022. This scoping review followed an established sequence of methodological steps (Arksey & O’Malley, 2005): (i) The research question was defined; (ii) studies/literature were identified and selected; (iii) resulting data were charted; (iv) results were collated, interpreted, summarized, and reported following an investigator triangulation strategy through Phases II to IV. We performed a search in four databases: Pubmed, ScienceDirect, JSTOR and the Cochrane Database of Systematic Reviews, using a combination of search terms. The following keywords were used: “ethical,” “breastfeeding,” “lactation,” and “ethics.” These search terms were specifically used to gather and capture the breadth of literature available in the topic area. In this study, the search strategy was based on WHO and UNICEF documents, so this manuscript uses predominantly cisnormative gendered language. ‘Chestfeeding’ and ‘bodyfeeding’ were not included in the search strategy. Search strategy applied to each database was “ethical AND breastfeeding AND lactation AND ethics.” The extent to which an article engaged explicitly with ethics-related issues determined whether it was considered to address “ethical issues.” For instance, an article mentioning cultural factors in implementing breastfeeding interventions may have addressed an ethical issue, but PubMed Advanced Search Builder may not have classified it as such since it did not explicitly mention “ethics.” Thus, this scoping review concentrated only on articles that met the inclusion criteria and could be accessed using the search strategy applied.

### Measurement

Since the primary objective of this scoping review was to describe the state of the literature, we did not conduct a quality appraisal of each included study, as is typically performed in systematic reviews. Instead, we analyzed all studies that met our inclusion criteria. Data was analyzed quantitatively and qualitatively. Quantitative analysis included a systematic count of the number of studies involving different: (1) study locations; (2) locations of authors; (3) topic areas; (4) methodological approaches; (5) intervention settings; and (6) key ethical issues. Qualitative analysis identified the key ethical themes and concepts reported in each study.

### Data Analysis

We used a data extraction spreadsheet to extract the following details: author names, year of publication, year of data collection, location of study and location of authors, study aims, topic area, research approach, sampling process, key findings, intervention settings, who carried out the research and key ethical issues of the study (Tables 1 and 2, and Supplemental Table 2). The first step was for SS to analyze all of the sample articles and present the emerging ethical themes in the articles, then SS discussed them with RV, NBA, and MH to build consensus. Each article was categorized and analyzed under six key themes: (1) respect for maternal autonomy and experiences, (2) counseling and informed consent process, (3) evidence and effectiveness of breastfeeding interventions, (4) ethical issues related to health communication, (5) ethical acceptability of financial incentives, and (6) children’s right to breastmilk.

**Table 1.** Characteristics of the Studies.

1st Author (year) Location (of the first author)	Study Aim	Sample	Data Sources	Data Collection	Trustworthiness
Shela Akbar Ali Hirani (2016) Canada	To analyze the concept of maternal autonomy in the context of BF and propose a clearer definition of the concept.	NA	PubMed,the Cumulative Index to Nursing and Allied Health Literature (CINAHL), Medline, and Google Scholar	Walker and Avant’s eight-stage approach	No detailed analysis of methodological approach.
Thierry Hurlimann (2017) Canada	To perform a scoping review which delineates and “maps” the range of ethical issues in nutrition-related public health interventions, and the range of the various fields in which they may arise.	*N*=169 articles	PubMed with Medical Subject Headings (MeSH) categories and Advanced Search Builder and in the Global Health Library	Arksey and O’Malley framework	Followed methodological approach as suggested by Arksey and O’Malley.
Katelin Hoskins (2019) USA	To systematically review and identify what is known about financial incentives directed to patients for health-related behavior change, assess how acceptability varies, and address which aspects and features of financial incentives are potentially acceptable and not acceptable, and why.	*N*=43 articles	Medline, Embase, Web of Science, Cumulative Index to Nursing and Allied Health Literature, PsycINFO, Sociological Abstracts, Scopus, Philosopher’s Index, Cochrane Library, and International Bibliography for Social Sciences	Preferred Reporting Items for Systematic Reviews (PRISMA) guidelines	PRISMA methodological approach.
Bodil Bø Våga (2014) Norway Study location (Tanzania)	To explore the applicability of the principle of informed choice in local health-care settings in Tanzania within prevention of mother-to-child transmission of HIV (PMTCT) programs in two hospitals in rural and semi-urban Tanzania through nurse counsellors’ and HIV-positive women’s experiences of infant feeding, counselling, and patient choice	Two hospitals: (1) church-run hospital in rural Tanzania, (2) Government hospital in semi-urban Tanzania. 76 HIV-positive women interviewed.	Observation, informal conversations (through home visits and local meetings), interviews (*N*=76) Follow-up interviews or observations: (*n*=22) Follow-up home visits: (*n*=18)	Field notes, audio recordings, verbatim transcriptions.	Followed a methodological approach.
Jane South (2014) UK	To critically discuss remuneration in the context of the management of lay health worker programs, reporting findings from a study of approaches to develop and support lay people in public health roles conducted in England, UK	Phase 1: Scoping Review (*n*=224) articles, 3 public hearings (n=3), 41 Register of projects Phase 2: Case studies (*n*=136).	Systematic scoping review of relevant and grey literature	Scientific databases, oral evidence and framework analysis, web-based registry with a data extraction form, audio recordings and verbatim transcriptions.	Followed a methodological approach.
Catherine M Fetherston (2012) Australia	To examine the competing principles involved in the bedsharing and BF debate, and arrive at a position constructed using ethical considerations.	NA / Not provided	Conceptual analysis	Conceptual analysis	Did not follow an established review strategy, rather, focused on conceptual discussion.
Rebecca Kukla (2006) USA	To closely and critically read the content and strategy of feeding advocacy campaign sponsored by the US State Department	NA / Not provided	Conceptual analysis	Conceptual analysis	Did not follow an established review strategy, rather, focused on conceptual discussion.
Sara Rosenthal (2006) Canada	To find out directly from women whether they were sufficiently counselled during their pregnancy about tests, birth planning, and overall labor and delivery expectations.	10 participants.	Semi-structured interview	Two-hour interviews; field notes	Followed a methodological approach and included reflective notes on the method.
R. Bennett (2007) UK	To considers the ethics of routine antenatal HIV testing and the role of informed consent.	NA / Not provided	Conceptual analysis	Conceptual analysis	Did not follow an established review strategy rather focused on conceptual discussion.
Sara E. Yeatman (2007) US	To address and critique the assumptions underlying a serostatus-based approach to behavior change and discuss the ethical consequences of transferring control of the decision to be tested from the individual to the provider	NA / not provided	Conceptual analysis	Conceptual analysis	Did not follow an established review strategy rather focused on conceptual discussion.
Rebecca Kukla (2008) Canada	To analyse reductive understanding of mothering, measuring mothering, and its implications	NA / Not provided	Conceptual analysis	Conceptual analysis	Did not follow an established review methodology, rather, focused on conceptual discussion.
Lise Rosendal Østergaard (2010) Denmark Study location Malawi	To explore challenges HIV positive women in Malawi face when they have to decide how to feed their infants.	28 participants	Observations and in-depth interviews	Audio recordings, verbatim transcription, fieldnotes	Followed a methodological approach along with reflective notes on the method.
Karleen D. Gribble (2010) Australia	To examine child’s right to breast milk	Two case studies: child protection authorities had interactions with a BF mother	Case study (n=2)	Reports, court documents	Did not follow any established review strategy, rather, focused on conceptual discussion.
Anne Barnhill (2015) USA	To examine the analogies and disanalogies with healthy eating policies to illuminate important ethical complexities of breast-feeding policy.	NA / Not provided	Conceptual analysis	Conceptual analysis	Did not follow an established review strategy, rather, focused on conceptual discussion.
Rebecca C H Brown (2015)	To explore Sandel’s corruption argument and consider its validity in the context of health incentives.	NA / Not provided	Conceptual analysis	Conceptual analysis	Did not follow an established review strategy, rather, focused on conceptual discussion.
Jessica Martucci (2018) USA	To critically examine the use of ’natural’ language in BF promotion by public health and medical bodies.	NA / Not provided	Conceptual analysis	Conceptual analysis	Did not follow an established review strategy, rather, focused on conceptual discussion
Joan B. Wolf (2007) USA	To critically analyse the National BF Awareness Campaign (NBAC).	NA / Not provided	Conceptual analysis	Conceptual analysis	Did not follow an established review strategy, rather, focused on conceptual discussion
Michele K. Griswold (2017) USA	To emphasize that BF provides ample evidence frame and BF as a critical component of health equity.	NA / Not provided	Conceptual analysis	Conceptual analysis	Did not follow an established review strategy, rather, focused on conceptual discussion
Jessica Nihlén Fahlquist (2011) Sweden	To use insights from the ethics of risk to critique current BF policy.	NA / Not provided	Conceptual analysis	Conceptual analysis	Did not follow an established review strategy, rather, focused on conceptual discussion
Erin N Taylor (2012) USA	To provide a new framework for understanding infant-feeding-related maternal guilt and shame, placing these in the context of feminist theoretical and psychological accounts of the emotions of self	NA / Not provided	Conceptual analysis	Conceptual analysis	Did not follow an established review strategy, rather, focused on conceptual discussion
Rhonda Shaw (2004) USA	To critique BF literature and policy	NA / Not provided	Conceptual analysis	Conceptual analysis	Did not follow an established review strategy, rather, focused on conceptual discussion.
Marielle S. Gross (2021) USA	To assess the evidence and ethical justification for current policy, with attention to pertinent racial and health disparities	Not provided	Pubmed, EMBASE, Cochrane, Web of Science and Google	Conceptual analysis Literature search of primary, secondary and grey literature;	Did not follow an established review methodology. Furthermore, focus was on conceptual discussion.
Ifeyinwa V. Asiodu (2021) USA	To critically review BF strategies and interventions, and argue for decolonizing BF research and clinical practice.	NA / Not provided	Conceptual analysis	Conceptual analysis	Did not follow an established review strategy, rather, focused on conceptual discussion
Vera K. Wilde (2021) Berlin	To critically argue that misconception of exclusive BF as natural and thus safe causes common and preventable harm to neonates.	NA / Not provided	Conceptual analysis	Conceptual analysis	Did not follow an established review methodology. Discussion of quality is insufficient.
Ingrid Nilsson (2022) Denmark	To explore needs, experiences and socio-cultural context of young and short-term educated mothers and their partners affecting BF duration and self-efficacy during pregnancy and the first months following birth.	14 participants	Interviews; focus group interviews; observations	Interviews; transcripts	Followed a methodological approach along with reflective notes on the method.
Kathryn L. MacKay (2021) Australia	To focus on 2 problematic aspects of British health-promotion campaigns regarding feeding children, particularly regarding BF and obesity.	NA / Not provided	Conceptual analysis	Conceptual analysis	Did not follow an established review strategy, rather, focused on conceptual discussion

*Note.* BF = breastfeeding; USA = United States of America; UK = United Kingdom; NA = not applicable; MeSH = Medical Subject Headings

**Table 2.** Key Topic Areas and Ethical Issues.

#^a^	1st Author (year)	Title	Intervention type^b^	Topic areas	Approach	Ethical Issues
1	Hirani (2016)	Concept Analysis of Maternal Autonomy in the Context of Breastfeeding	Educational	Normative understanding of motherhood and breastfeeding	Conceptual and ethical analysis	Respect for maternal autonomy and mother’s experiences
2	Hurlimann (2017)	Ethical issues in the development and implementation of nutrition-related public health policies and interventions: A scoping review	Educational Environmental	Ethical issues in infant feeding research	Scoping review	Evidence and effectiveness of breastfeeding interventions
3	Hoskins (2019)	Acceptability of financial incentives for health-related behavior change: An updated systematic review	Educational Environmental	Financial incentives	Scoping review	Ethical acceptability of financial incentives
4	Våga (2014)	Reflections on informed choice in resource-poor settings: The case of infant feeding counselling in PMTCT programmes in Tanzania	Educational	Counselling, decision making and social context	Ethnographic research	Respect for maternal autonomy and mother’s experiences
5	South (2014)	Rewarding altruism: Addressing the issue of payments for volunteers in public health initiatives	Educational	Financial incentives	Scoping review	Ethical acceptability of financial incentives
6	Fetherston (2012)	Analysis of the ethical issues in the breastfeeding and bedsharing debate	Educational	Normative understanding of motherhood and breastfeeding	Conceptual and ethical analysis	Evidence and effectiveness of breastfeeding interventions
7	Kukla (2006)	Ethics and Ideology in Breastfeeding Advocacy Campaigns	Educational Environmental	Normative understanding of motherhood and breastfeeding	Conceptual and ethical analysis	Respect for maternal autonomy and mother’s experiences
8	Rosenthal (2006)	Socioethical Issues in Hospital Birth: Troubling Tales from a Canadian Sample	Educational	Counselling, decision making and social context	Ethnographic research	Counselling and informed consent process
9	Bennett (2007)	Routine Antenatal HIV Testing and Informed Consent: An Unworkable Marriage?	Educational	Counselling, decision making and social context	Conceptual and ethical analysis	Counselling and informed consent process
10	Yeatman (2007)	Ethical and Public Health Considerations in HIV Counseling and Testing: Policy Implications	Educational	Counselling, decision making and social context	Conceptual and ethical analysis	Counselling and informed consent process
11	Kukla (2008)	Measuring Mothering	Educational	Normative understanding of motherhood and breastfeeding	Conceptual and ethical analysis	Respect for maternal autonomy and mother’s experiences
12	Østergaard (2010)	"They call our children „Nevirapine Babies?”: A Qualitative Study about Exclusive Breastfeeding among HIV Positive Mothers in Malawi	Educational	Counselling, decision making and social context	Conceptual and ethical analysis	Counselling and informed consent process
13	Gribble (2010)	Rights of Children in Relation to Breastfeeding in Child Protection Cases	Educational	Children’s right to breastfeeding	Ethnographic research	Children’s right to breastmilk
14	Barnhill (2015)	Latch On or Back Off? Public Health, Choice, and the Ethics of Breast-Feeding Promotion Campaigns	Educational Environmental	Normative understanding of motherhood and breastfeeding	Conceptual and ethical analysis	Respect for maternal autonomy and mother’s experiences
15	Brown (2015)	Social values and the corruption argument against financial incentives for healthy behaviour	Educational Environmental	Financial incentives	Conceptual and ethical analysis	Ethical acceptability of financial incentives
16	Martucci (2018)	Examining the use of ‘natural’ in breastfeeding promotion: ethical and practical concerns	Educational	Breastfeeding promotions	Conceptual and ethical analysis	Respect for maternal autonomy and mother’s experiences
17	Wolf (2007)	Is Breast Really Best? Risk and Total Motherhood in the National Breastfeeding Awareness Campaign	Educational Environmental	Breastfeeding promotions	Conceptual and ethical analysis	Ethics of health communication
18	Griswold (2017)	Reframing the Context of the Breastfeeding Narrative: A Critical Opportunity for Health Equity Through Evidence-Based Advocacy	Educational Environmental	Breastfeeding promotions	Conceptual and ethical analysis	Ethics of health communication
19	Fahlquist (2011)	Ethical Problems with Information on Infant Feeding in Developed Countries	Educational Environmental	Breastfeeding promotions	Conceptual and ethical analysis	Ethics of health communication
20	Taylor (2012)	For Shame: Feminism, Breastfeeding Advocacy, and Maternal Guilt	Educational	Normative understanding of motherhood and breastfeeding	Conceptual and ethical analysis	Respect for maternal autonomy and mother’s experiences
21	Shaw (2004)	Performing Breastfeeding: Embodiment, Ethics and the Maternal Subject	Educational	Normative understanding of motherhood and breastfeeding	Conceptual and ethical analysis	Respect for maternal autonomy and mother’s experiences
22	Gross (2021)	Breastfeeding with HIV: An Evidence-Based Case for New Policy	Educational	Counselling, decision making and social context	Conceptual and ethical analysis	Counselling and informed consent process
23	Asiodu (2021)	Achieving Breastfeeding Equity and Justice in Black Communities: Past, Present, and Future.	Environmental	Decolonising breastfeeding	Conceptual and ethical analysis	Respect for maternal autonomy and mother’s experiences
24	Wilde (2021)	Breastfeeding Insufficiencies: Common and Preventable Harm to Neonates.	Environmental	Ethical issues in infant feeding research	Conceptual and ethical analysis	Evidence and effectiveness of breastfeeding interventions
25	Nilsson (2022)	Breastfeeding trajectories of young and short-term educated mothers and their partners; experiences of a journey facing tailwind and headwind.	Educational Environmental	Counselling, decision making and social context	Qualitative/Ethnographic research	Respect for maternal autonomy and mother’s experiences
26	MacKay (2021)	Mothers: The Invisible Instruments of Health Promotion	Environmental	Normative understanding of motherhood and breastfeeding	Conceptual and ethical analysis	Respect for maternal autonomy and mother’s experiences

*Note.* ^a^ indicates study number as they are referenced in Figures 1 and 2. ^b^An educational intervention aims to change individual behaviors and an environmental intervention seeks to change the social and environmental conditions that encourage, require, or reinforce behaviors that are either beneficial or harmful to health. Adopted from Buchanan (2019).

## Results

Some of the major ethical issues discussed in the sample articles (*N* = 26) were on the normative assumptions of motherhood, maternal autonomy, and breastfeeding choice (Asiodu et al., 2021; Fetherston & Leach, 2012; Hirani & Olson, 2016; Kukla, 2006, 2008; MacKay, 2021; Østergaard & Bula, 2010; Shaw, 2004; Taylor & Wallace, 2012; Våga et al., 2014). A theme of information disclosure, balancing risks and benefits, and counseling practices also arose (Bennett, 2007; Gross et al., 2019; Nilsson et al., 2022; Rosenthal, 2006; Våga et al., 2014; Yeatman, 2007). Stigma and social context was discussed (Bennett, 2007; Nilsson et al., 2022; Yeatman, 2007). Several articles covered the ethics of health communication, particularly in breastfeeding campaigns (Barnhill & Morain, 2015; Fahlquist & Roeser, 2011; Griswold, 2017; Kukla, 2006; Martucci & Barnhill, 2018; Taylor & Wallace, 2012; Wolf, 2007), and the ethical acceptability of financial incentives in breastfeeding interventions was also discussed (Brown, 2017; Hoskins et al., 2019; South et al., 2014).

**Figure 2.**
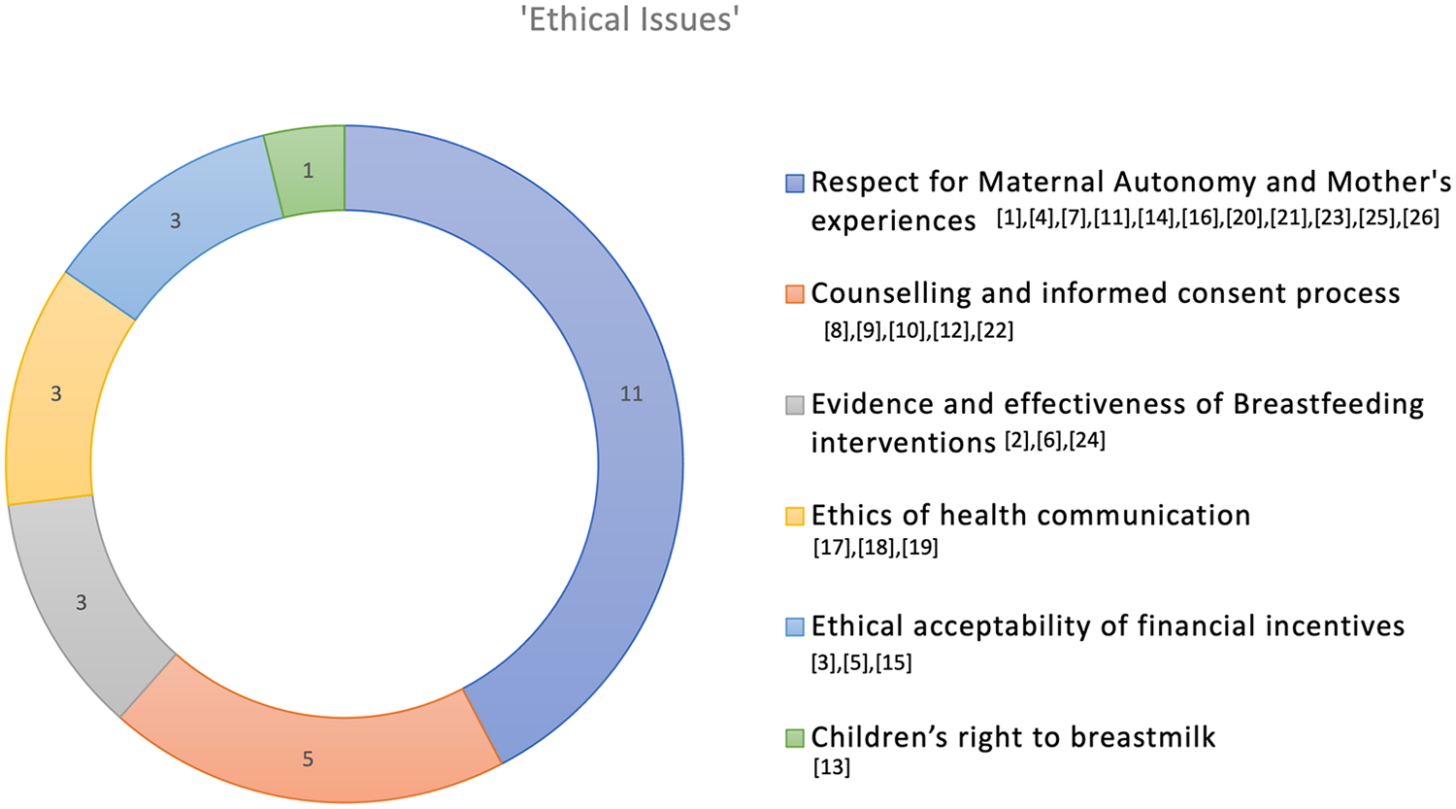
Ethical Issues Identified in Study Articles. *Note.* Numbers in brackets represent articles identified in Table 2.

Some articles included discussions on decolonizing breastfeeding research and clinical practice, particularly to address the health needs of women from Black, Indigenous and People of Color (BIPOC) communities (Asiodu et al., 2021). Other articles covered issues relating to autonomy, and scientific consensus around breastfeeding in relation to bedsharing and neonates (Fetherston & Leach, 2012; Wilde, 2021), and children’s right to breastfeeding and nutrition (Gribble & Gallagher, 2014; Wilde, 2021). The ethical issues discussed in these articles could sometimes overlap as the authors reflected on intertwined ethical concepts. Based on the scoping review, Figure 2 illustrates the key emerging ethical issues, where the dominant focus of each article was categorized as interpreted by the authors.

**Figure 3.**
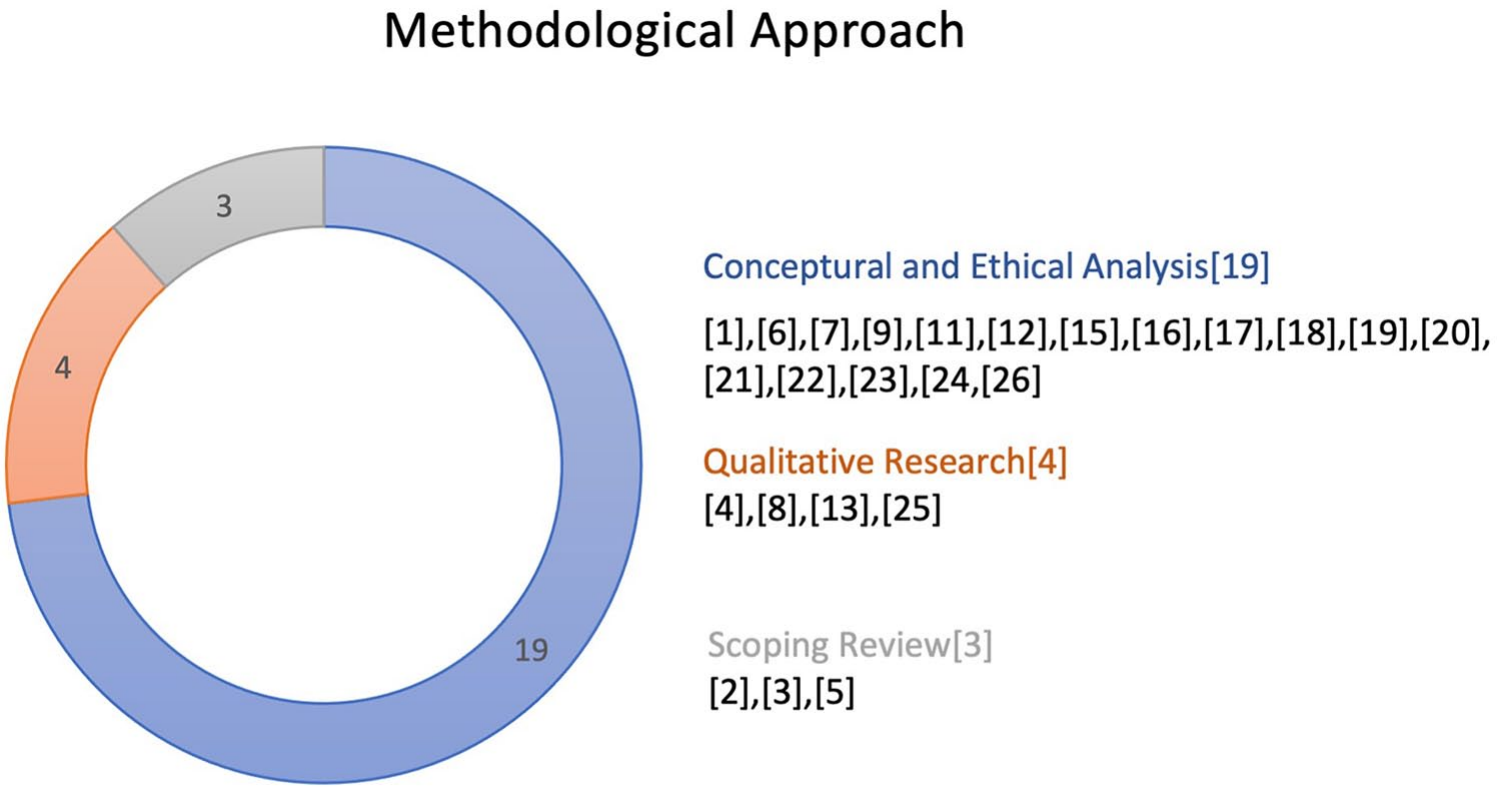
Methodological Approach Identified in Articles. *Note.* Numbers in brackets represent articles as listed in Table 2.

The dominant methodological approach for engaging with the ethical issues of breastfeeding interventions was a conceptual and ethical analysis, as found in 19 articles (Asiodu et al., 2021; Barnhill & Morain, 2015; Bennett, 2007; Brown, 2017; Fahlquist & Roeser, 2011; Fetherston & Leach, 2012; Gribble & Gallagher, 2014; Griswold, 2017; Gross et al., 2019; Hirani & Olson, 2016; Kukla, 2006, 2008; MacKay, 2021; Martucci & Barnhill, 2018; Shaw, 2004; Taylor & Wallace, 2012; Wilde, 2021; Wolf, 2007; Yeatman, 2007). There were three articles in the form of scoping reviews that addressed some aspects of breastfeeding as part of a broader focus on nutrition-related public health policies. Authors of one of the scoping review articles identified common ethical issues, including infants’ best interests, ethical challenges in public–private partnerships (e.g., conflicts of interest), and marketing, advertisement, and labeling of human milk substitutes in breastfeeding programs (Hurlimann et al., 2017). In that article, authors mapped the ethical issues in nutrition-related public health interventions, where breastfeeding was one of many other topics mapped (i.e., food security, food safety, nutrition, public health ethics). Other scoping reviews focused on the ethical acceptability of financial incentives (Hoskins et al., 2019; South et al., 2014). There were four articles categorized as ethnographic research (Nilsson et al., 2022; Østergaard & Bula, 2010; Rosenthal, 2006; Våga et al., 2014), as presented in Figure 3.

## Discussion

Infant feeding, particularly breastfeeding, has been given a key priority in achieving global health standards (Rollins et al., 2016; The Lancet, 2017), with many national and international programs endorsing exclusive breastfeeding for the first 6 months (Lee and Binns, 2019; WHO, 2008). Most breastfeeding policies and programs have employed various interventions to increase the awareness of breastfeeding and increase breastfeeding rates (Haroon et al., 2013; Kim et al., 2018). In this scoping review we presented the key ethical issues highlighted in infant feeding within the clinical, social sciences, and humanities literature. A key observation from the results (Table 2) was that most interventions focused on prevention through educational interventions, counseling, and education materials, but not environmental interventions to support lactating persons.

### Normative Assumptions and Breastfeeding Interventions

The literature has been presented as dichotomous, focusing on populations versus individuals when discussing the ethical implications of public health interventions. Results from this scoping review showed that breastfeeding interventions could not be neatly categorized into population or individual levels (see Table 2 and Supplemental Table 2). In the paper by Hirani and Olson (2016), the concept of maternal autonomy was critically analyzed from a maternal and child health context. The authors emphasized the importance of maternal autonomy in relation to both the mother’s and child’s health, from a holistic perspective. Within this definition they included maternal agency and ethical reasoning, as well as maternal independence and resource control. Therefore, they suggested that any breastfeeding interventions needed to account for the availability of support and the nature of the setting and available feeding alternatives. Additionally, as discussed by MacKay (2021), a mother’s agency can be undermined within breastfeeding campaigns and instrumentalized toward achieving goals in public health campaigns. Thus, they emphasized that breastfeeding equally affects maternal and child health, and that it is therefore imperative for stakeholders to facilitate maternal autonomy by taking into consideration the larger context.

Most infant feeding literature has highlighted the dominant models adopted in breastfeeding educational interventions—the maternalist and the medical models (Blum, 1993; Lee, 2018; Wall, 2001). The maternalist model emphasizes motherhood and the embodied connection between a mother and child, whereas the medical model focuses on benefits of “breastmilk” and explicitly educates mothers to provide milk for a child’s development and growth (Blum, 1993). Both of these models have been adopted into most breastfeeding educational interventions. This can be observed in the articles analyzed in breastfeeding promotions (Martucci & Barnhill, 2018; Wolf, 2007) or counseling (Yeatman, 2007). The breastfeeding discourse was generally placed within population health; however, since breastfeeding was constituted as a maternal subject, it also belonged to the individual health level. There are many sociological and anthropological studies which illustrate how breastfeeding intimately connects women’s bodies to infant bodies in relation to embodiment, sexuality, reproduction, and other policy issues, like women’s employment and workplace access (Lee, 2018; Martucci & Barnhill, 2018; Murphy, 1999; Wall, 2001).

Any policy intervention that involves multiple stakeholders has normative assumptions about motherhood. This underlying normative dimension was highlighted in many articles we analyzed, engaging the concepts of a “good mother,” “informed choice,” and “maternal autonomy” (Hirani & Olson, 2016; Kukla, 2006, 2008; MacKay, 2021; Shaw, 2004; Taylor & Wallace, 2012; Våga et al., 2014). Discussions of these ethical issues suggested going beyond the normative assumptions in breastfeeding interventions and accounting for maternal experiences in designing and implementing policies and programs (Griswold, 2017). Furthermore, Asiodu et al. (2021) found that decolonizing breastfeeding research and clinical practice was necessary to address the health needs of women from ethnically marginalized backgrounds. The authors emphasized how important it was for infant feeding research to be inclusive, and not exclude people on the basis of their racial, ethnic, or cultural backgrounds, in order to increase breastfeeding rates among Black women (Asiodu et al., 2021).

The Lancet’s seminal series on breastfeeding, which covered the effectiveness of promotion interventions, highlighted the need to shift the focus on women’s responsibility in breastfeeding to a broader societal responsibility to facilitate women’s choice to breastfeed in policy interventions and programs (Rollins et al., 2016; Victora et al., 2016). According to the authors of one of the recent Lancet series articles (Baker et al., 2023), health systems have not adequately protected, promoted, and supported breastfeeding. The authors drew attention to gendered and biomedical power structures denying women centered care, as well as the larger political economy, which promotes acceptance, commercialization, commercial influence, and conflicts of interest. They highlight the lack of recognition of breastfeeding as care work as one of the primary factors in low breastfeeding rates, given that this work is typically devalued in society. In another article (Pérez-Escamilla et al., 2023), it was pointed out that effective breastfeeding interventions require a socioecological model in a market driven world. They also stress that breastfeeding should not be solely women’s responsibility. These authors pointed out, as did many scholars in the discipline of reproductive justice (Labbok et al., 2008; Lee, 2019; Pérez-Escamilla et al., 2023; Smith, 2018), that acknowledging gender inequalities in society was an essential component of fostering collective societal solutions (Pérez-Escamilla et al., 2023). Most of the articles in our scoping review highlighted this need to support breastfeeding mothers who were affected by the larger historical, social, cultural, and economic context in which they lived. In situations in which health disparities are rooted in social inequalities, interventions and health research based only on biomedical models has been ineffective. It is necessary to critically engage with the context of breastfeeding. Thus, to summarize, the authors of these articles urged that breastfeeding policies and interventions acknowledge a broader structure, including political and societal factors, while promoting breastfeeding by recognizing mothers’ and lactating persons’ experiences.

### Breastfeeding Interventions, Practice, and Social Context

Health promotion communication interventions typically raise ethical issues targeting individuals’ values, beliefs, and lifestyles (Carter et al., 2012; Guttman, 2017). The key ethical concerns identified from breastfeeding promotion intervention studies were related to the idea of the breast as “natural” narrative, privacy, data protection, stigma, labeling, informed choice, and consent processes (Bennett, 2007; Fahlquist & Roeser, 2011; Griswold, 2017; Knaak, 2006; Kukla, 2006; MacKay, 2021; Martucci & Barnhill, 2018; Nilsson et al., 2022; Taylor & Wallace, 2012). These issues emphasized the significance of maternal autonomy and the need to account for mothers’ experiences within the breastfeeding discourse. While there were rich discussions around the ethics of health communication to facilitate ethical interventions for policymakers or stakeholders (Carter et al., 2012; Faden, 1987; Guttman, 2017), these articles highlighted the gap in adopting ethical frameworks specifically for breastfeeding interventions. Thus, identifying and critically assessing ethical issues in the design and implementation processes is imperative to breastfeeding promotion interventions.

Articles from our sample also illustrated the ethical acceptability of financial incentives in breastfeeding interventions (Brown, 2017; Hoskins et al., 2019; South et al., 2014). While some scholars dispute the quality of breastfeeding evidence (Colen & Ramey, 2014; Kramer et al., 2007), many national and international organizations, including the WHO and UNICEF, recommend exclusive breastfeeding for the first 6 months of life and continued breastfeeding for at least 1 year (WHO & UNICEF, 2012). A critical gap exists between international guidelines and breastfeeding prevalence rates due to various social determinants (Hoskins & Schmidt, 2021; Moran et al., 2015). At the time these recommendations were made, breastfeeding rates were relatively low globally, possibly due to the nature of breastfeeding and its barriers, that is, difficulty with lactation, lack of family and system support, lack of information, economic costs, cultural and social norms, and the shifting identities of motherhood (Jones et al., 2015; Patil et al., 2020). Hoskins and Schmidt (2021) suggest that implementing carefully designed financial incentives for breastfeeding interventions can be considered as a potential tool to increase breastfeeding rates.

In our sample, the use of financial incentives to promote behavioral change, particularly in the context of breastfeeding, led to a discussion of ethical concerns—shame, fairness, nudging, coercion, and personal responsibility—which once again targeted mothers’ decisions. A recent study brought together key ethical issues around financial incentives in breastfeeding promotions (Hoskins & Schmidt, 2021). The authors suggested a variety of infant feeding choice architectures for carefully designing ethically justified financial incentives. They also emphasized the need to further investigate ethical concerns related to decision-making in disadvantaged and vulnerable populations. This illustrates an important aspect to consider when targeting breastfeeding interventions toward maternal decisions for the purpose of achieving maternal and child health goals.

Our review also identified other key ethical concerns in breastfeeding interventions including reducing stigma and ensuring information disclosure and appropriate counseling practices (Bennett, 2007; Gostin & Kavanagh, 2019; Nilsson et al., 2022; Yeatman, 2007). Particularly among women diagnosed with HIV, some of these studies emphasized the importance of counseling and the informed consent process when facilitating shared decision-making to promote breastfeeding (Bennett, 2007; Yeatman, 2007). Gross et al. (2019) used justice-based arguments to ensure that women living with HIV receive donor or formula milk if breastfeeding is not optimal within particular contexts. Using ethical principles of autonomy, harm reduction, and health inequities, they emphasized the importance of revising blanket decisions against breastfeeding for women living with HIV in policy decisions and guidelines. The authors of these articles cautioned that counseling and information disclosure processes, as well as any educational interventions, needed to be sensitive to language and messaging to avoid affecting maternal agency and stigmatizing vulnerable groups.

Other articles from this scoping review focused on children’s rights to breastfeed (Gribble & Gallagher, 2014), where the importance of a child’s healthy development was significant, as well as the obligations of a mother towards her child. There were also articles examining bed sharing with infants (Fetherston & Leach, 2012), applying an ethical framework based on utility, evidence-based, effective action, fairness, accountability, burdens, costs, and community acceptance (Baum et al., 2007). According to Wilde (2021), inadequate breastfeeding practices caused common and preventable harms to neonates. Based on this framework, ethical issues were analyzed in policy recommendations for breastfeeding and bedsharing decisions (Fetherston & Leach, 2012) suggesting that current evidence warrants fundamental policy changes in infant feeding.

All these studies reflected the need for ethical assessment and guidance while designing and implementing breastfeeding interventions so that the economic, social, cultural and historical contexts are fully captured and addressed. Pérez-Escamilla et al. (2023) described how the commercial milk formula industry exploited vulnerable parents and outlined the industry’s violation of the WHO’s International Code on the Marketing of Breast-milk Substitutes. Breastfeeding interventions could be facilitated through ethical frameworks in public health practice (Baum et al., 2007; Buchanan, 2019; Gopichandran et al., 2016; Kass, 2001; Marckmann et al., 2015). Due to the distinctive nature of breastfeeding, while these frameworks are helpful, it is necessary to carefully analyze ethical tensions as they are being developed to inform breastfeeding guidelines and frameworks. The local contexts need to be an integral part of any ethical review before design and implementation begin.

Stakeholders, policymakers and decision-makers have been left without clear guidance while designing and implementing breastfeeding interventions. It is necessary to justify interventions from a systematic ethical assessment considering both population and individual levels, and to assess the overall impact on the targeted community. As reflected in our results, most authors addressed how lactating persons’ experiences within historical, cultural, and social contexts should be accounted for in guiding and determining health policy and intervention decisions. While scientific bodies had evidentiary considerations for adopting a child-centered approach to breastfeeding, ethical assessment of unintended consequences of interventions, a threat to maternal autonomy, shame, guilt, or stigma, must also be considered (Leeming, 2018; Taylor & Wallace, 2012; 5; Tomori et al., 2016). In addition to ensuring professional standards, an ethical assessment of the risks and benefits of health interventions will help to achieve the intended public goal and provide more relevant and impactful interventions.

### Limitations

Other than the limitations described in the Methods section, we acknowledge that our primary focus was on studies or articles that explicitly engaged with ethical issues in breastfeeding and lactation interventions. As a result of the search strategy used in this study, the manuscript contained cisnormative gendered language and focused mostly on breastfeeding literature and mothers’ experiences and choices. A more inclusive examination in the literature would have included lactation feeding practices among transgender and gender non-conforming (TGNC) communities, as well as the wider LGBTQIA+ community. In this scoping review, ethical concepts and values were analyzed interpretively, and researchers acknowledge and embrace iterative and interpretive data analysis.

## Conclusion

This scoping review provided an overview of ethical issues discussed in the literature in the context of breastfeeding and lactation interventions. Health professionals and policy makers need systematic guidance through the broad array of ethical issues as they develop and implement ethical interventions. To resolve the ethical tensions between respecting women’s choices and experiences, and public health goals regarding infant nutrition and breastfeeding, it will be important to consider respective cultural, social, political and economic contexts and move beyond the dichotomous notion of individual health versus population health (private vs. public, or agency vs. structure). These articles covered ethical issues that suggest we examine interventions that support vulnerable lactating persons and infants before implementing policies and practices to achieve breastfeeding equity by acknowledging systemic social and gender inequities. In order for policy makers, implementers, and public health officials to develop targeted ethical frameworks and guidance, additional research is necessary to better understand the ethical issues associated with breastfeeding interventions.

## Supplemental Material

sj-xlsx-1-jhl-10.1177_08903344231215073 – Supplemental material for Ethical Issues in Breastfeeding and Lactation Interventions: A Scoping Review Click here for additional data file. Supplemental material, sj-xlsx-1-jhl-10.1177_08903344231215073 for Ethical Issues in Breastfeeding and Lactation Interventions: A Scoping Review by Supriya Subramani, Rasita Vinay, Julian W. März, Michaela Hefti and Nikola Biller-Andorno in Journal of Human Lactation

sj-xlsx-2-jhl-10.1177_08903344231215073 – Supplemental material for Ethical Issues in Breastfeeding and Lactation Interventions: A Scoping Review Click here for additional data file. Supplemental material, sj-xlsx-2-jhl-10.1177_08903344231215073 for Ethical Issues in Breastfeeding and Lactation Interventions: A Scoping Review by Supriya Subramani, Rasita Vinay, Julian W. März, Michaela Hefti and Nikola Biller-Andorno in Journal of Human Lactation
